# The Establishment of Genetically Engineered Canola Populations in the U.S.

**DOI:** 10.1371/journal.pone.0025736

**Published:** 2011-10-05

**Authors:** Meredith G. Schafer, Andrew A. Ross, Jason P. Londo, Connie A. Burdick, E. Henry Lee, Steven E. Travers, Peter K. Van de Water, Cynthia L. Sagers

**Affiliations:** 1 Department of Biological Sciences, University of Arkansas, Fayetteville, Arkansas, United States of America; 2 Department of Biological Sciences, North Dakota State University, Fargo, North Dakota, United States of America; 3 Western Ecology Division, National Health and Environmental Effects Research Laboratory, U.S. Environmental Protection Agency, Corvallis, Oregon, United States of America; 4 Earth and Environmental Sciences Department, California State University, Fresno, California, United States of America; Cinvestav, Mexico

## Abstract

Concerns regarding the commercial release of genetically engineered (GE) crops include naturalization, introgression to sexually compatible relatives and the transfer of beneficial traits to native and weedy species through hybridization. To date there have been few documented reports of escape leading some researchers to question the environmental risks of biotech products. In this study we conducted a systematic roadside survey of canola (*Brassica napus)* populations growing outside of cultivation in North Dakota, USA, the dominant canola growing region in the U.S. We document the presence of two escaped, transgenic genotypes, as well as non-GE canola, and provide evidence of novel combinations of transgenic forms in the wild. Our results demonstrate that feral populations are large and widespread. Moreover, flowering times of escaped populations, as well as the fertile condition of the majority of collections suggest that these populations are established and persistent outside of cultivation.

## Introduction

Crop and forage species now cover more than one quarter of the Earth's land surface [1], but the ecological and evolutionary influences of agricultural species on native and weedy plants have been difficult to measure. The commercial release of GE crops has provided novel genetic markers to track crop-to-weed gene flow [2], [3] raising both awareness of the difficulties of transgene confinement and concerns about the ecological consequences of transgenes in the environment [4], [5]. Genetically engineered varieties could influence the population ecology of wild species by introducing novel, beneficial traits, or lead to detrimental effects such as extirpation of native alleles or declines of natural populations [6]. The escape of crops or crop alleles is no longer in doubt [7], but reports of transgene escape are few and are limited in the U.S. to the case of creeping bentgrass, *Agrostis stolonifera* (Poaceae), from a field trial in central Oregon, USA [8], [9]. Given that biotech crops cover more than 130Mha globally [10], the rarity of reported escapes has led some to question the environmental risks of genetically engineered crops [11], [12].

Canola (*Brassica napus* L. (Brassicaceae)) is an oilseed crop grown on approximately 31Mha globally [13]. *Brassica napus*, an allotetraploid formed by the hybridization of *B. rapa* L. and *B. oleraceae* L., is sexually compatible with more than 15 other mustard species [14], a number of which are considered noxious weeds [15]. Canola cultivars engineered for glyphosate and glufosinate herbicide resistance escaped cultivation shortly after their unconditional commercial release in Canada in 1995 [16] and more recent research has documented widespread escape and persistence of transgenic canola in Canadian roadside populations [17], [18]. Since these discoveries, feral canola populations or non-engineered populations expressing biotech traits have been reported from Great Britain, France, Australia and Japan [2], [3], [19]–[21]. In the U.S., GE canola was first approved for commercial release in 1998 and now most (>90%) of the acreage planted in the U.S. is genetically engineered for herbicide resistance [10].

The objective of this study was to document the extent of feral canola populations in North Dakota, the dominant canola growing region of the United States. We used roadside surveys and commercially available test strips evaluate the distribution of transgenic canola growing outside of cultivation in the U.S.

## Materials and Methods

We conducted systematic roadside surveys to quantify the presence and abundance of feral GE and non-GE canola populations in North Dakota, USA, beginning 4 June and continuing through 23 July 2010. Field crews established east-west transects on major roads throughout the state. A 1×50 m quadrat was established every 8.05 km (5 miles) of roadway on one or both sides of the road, where traffic permitted, in which all identifiable *B. napus* plants were counted. We drove a total of 5600 km and sampled 63.1 km of roadside habitats (1.1% of the distance driven). Sampling was conducted early in the summer prior to the onset of flowering of cultivated canola. When canola was present at a sampling site, one randomly selected plant was collected, photographed and archived as a voucher specimen. Leaf fragments from voucher specimens were tested for the presence of CP4 EPSPS protein (confers tolerance to glyphosate herbicide) and PAT protein (confers tolerance to glufosinate herbicide) with TraitChek™ immunological lateral flow test strips (Strategic Diagnostics, Inc., Newark, DE). Previous studies have demonstrated the utility of the lateral flow strips in detecting the expression of transgenes from field samples [8], [22]. Test strips are not available for a third, non-GE resistance trait, resistance to Clearfield™ herbicide, which comprises approximately 10% of the canola grown in the region (R Beneda, pers comm). At random intervals, single plants were tested with multiple test strips to assure that test results were repeatable and reliable. No failures were detected during the course of the study. To determine if populations of escaped canola are composed of multiple genotypes, multiple plants were sampled and tested for the presence of CP4 EPSPS or PAT proteins at 9 randomly selected, large canola populations Test strips and plant voucher specimens are archived at the University of Arkansas. GPS locations and transgene state values for each collected plant are available in Table S1.

## Results

**Figure 1:**
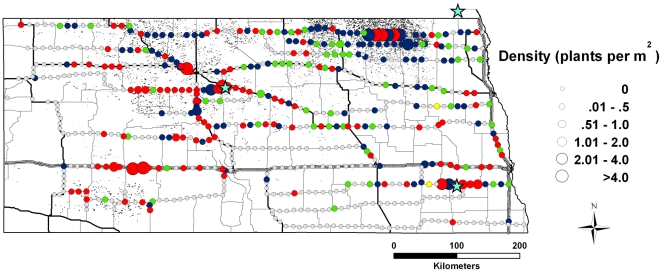
Distribution and density of feral canola populations in North Dakota road surveys (2010). Circles indicate locations of sampling sites; diameter of circle indicates plant density; gray circles indicate no canola present. The presence of genetically engineered protein in the vouchered specimen is shown by color: red – glyphosate resistance; blue – glufosinate resistance; yellow – dual resistance traits; green – non-transgenic. Canola fields are indicated by stippling based on 2009 USDA National Agricultural Statistics Service report (http://www.nass.usda.gov/Statistics_by_Subject/index.php?sector=CROPS). Stars show the locations of oilseed processing plants (3). Solid lines illustrate interstate, state and county highways.

The escape of GE *B. napus* in North Dakota is extensive (Fig. 1). *Brassica napus* was present at 45% (288/634) of the road survey sampling sites. Of those, 80% (231/288) expressed at least one transgene: 41% (117/288) were positive for only CP4 EPSPS (glyphosate resistance); 39% (112/288) were positive for only PAT (glufosinate resistance); and 0.7% (2/288) expressed both forms of herbicide resistance, a phenotype not produced by seed companies (Table 1). Densities of *B. napus* plants at collection sites ranged from 0 to 30 plants m^−2^ with an average of 0.3 plants m^−2^. Among the archived specimens, 86.8% were sexually mature varying in developmental stage from flower bud to mature fruit with seeds. At the time of roadside sampling, in-field canola was non-flowering having matured to the 4-leaf to pre-bolting stage (JPL pers. obs.). This striking difference in flowering phenology suggests that flowering canola in roadside habitats may have originated from the previous generation's seed bank rather than from seed spill during the current growing season.

**Table 1:** Distribution of transgenic and non-transgenic canola in North Dakota transects.

	# of sites	Percent
Total transects	634	
Canola present	288	0.454
Transgenic	231	0.802
Liberty Link+	112	0.389
Roundup Ready+	117	0.406
LL+ and RR+	2	0.007
Non-Transgenic		
Null	57	0.198

Populations of transgenic canola were denser along major transport routes, at construction sites and in regions of intense canola cultivation (Fig. 1). At a finer scale, feral populations appeared denser at junctions between major roadways, access points to crop fields and bridges, and intersections of roadways with railway crossings. At these sites, seed spill during transport is a likely mechanism for the escape of transgenic canola. Nonetheless, feral *B. napus* plants were occasionally found at remote locations far from canola production, transportation, or processing facilities. Populations were also observed at roadsides that had recently been mowed or treated with herbicide. Although our sampling protocol stipulated that a single plant be tested at each collection site, multiple sampling of additional plants revealed a mix of both herbicide resistant phenotypes, or a mix of herbicide resistant and vulnerable phenotypes in all randomly-tested large populations (Table S1).

## Discussion

To date there have been relatively few reports of the escape from cultivation of genetically engineered varieties leading some researchers to discount the environmental risks of biotech crops. Concurrently, public demonstrations have led to a consumer backlash against genetically engineered foods. A first step toward understanding the environmental impact of biotech crops is to identify the incidence and extent of their escape from cultivation. We conducted this study to document feral populations of genetically engineered canola and to evaluate potential mechanisms of persistence outside of crop fields.

The escape of canola from cultivation is not particularly surprising. *Brassica napus* is thought to have been domesticated very recently, in the last 300–400 years [23]. As a consequence, “wild” traits, such as seed shattering and partial seed dormancy, are still expressed in commercial canola and may contribute to escape from cultivation. For example, up to 30% of a seed crop may be lost each year by shattering during harvest [24] and canola seeds may remain dormant for up to three years [25]. The combined effects of seed loss on harvest and seed dormancy rapidly stock the soil seed bank, which can lead to frequent re-seeding of marginal soils [17].

Surprising from our study is the widespread distribution of feral canola outside of cultivated areas both near and far from cultivated fields over much of North Dakota and the likely persistence of these populations beyond single years. Additionally, these populations occur both in habitats with selection pressure (e.g., roadsides sprayed with glyphosate) and also in habitats without obvious selection pressure. Although canola cultivation in North Dakota occurs primarily in the northeastern counties, we identified transgenic canola populations in parts of North Dakota with little or no known canola production. Our results suggest a number of routes by which canola plants may be introduced to the wild. Feral canola populations were found in high densities along major trucking routes but not smaller tributaries suggesting that feral canola populations are established by seed spill. Similar results have been reported in studies of feral canola in Canada [17], [18]. The mixture of phenotypes that we found in 9 large populations, further suggests that multiple seed spills or dispersal events can occur at a given location. In addition, we identified large, continuous populations of feral transgenic canola (population IDs 215–216) growing on fill dirt at highway construction zones that clearly did not result from seed shatter or seed spill (JPL pers. obs.). We suggest that canola may colonize repositories of fill dirt and rapidly establish a soil seed bank. The movement of contaminated fill dirt to remote construction sites provides an additional mechanism for the dispersal of transgenic canola far beyond field margins.

Movement by transport is likely to explain the current distribution of feral canola populations in North Dakota, but re-seeding by fertile plants further contributes to population persistence. Our evidence that these populations persist outside of cultivation includes the striking difference in flowering phenology between feral and commercial populations. Flowering times differed by approximately four weeks, indicating that field and feral populations originated from different sources. Further evidence for persistence is found in our statewide collections of fertile plants with viable seeds. Metapopulation dynamics by which feral populations are fed by seed transport but supplemented by *in situ* seed production are likely at play here as described by [18] for feral canola populations in Canada.

The occurrence of novel resistance phenotypes may provide additional evidence that these populations can persist outside of cultivation. When transgenic resistance genotypes grow in sympatry, varieties may hybridize to create novel combinations of traits, as we found at two locations. Because resistance to multiple herbicides has not been commercially developed in canola, the discovery of “stacked” traits in feral canola plants is evidence that biotech varieties have hybridized. Hybridization could possibly have occurred by pollen flow between fields of transgenic canola varieties, followed by seed spill along roadsides. Alternatively, hybridization could have occurred by pollen movement among resistant phenotypes within roadside populations, because feral populations were frequently found to include multiple phenotypes, or by flow of transgenic pollen from other feral populations or crop fields. By whatever mechanism, hybridization among genetically engineered varieties is not uncommon. Although we sampled a relatively small number of plants (N = 288) from a small percentage of the total potential habitat along roadways in North Dakota (1.1%), we nonetheless identified two individuals expressing novel stacked traits (0.7%). Furthermore, the incidence of crop-crop hybridization is under-sampled in this survey because test strips for a third commercial form of herbicide resistant canola, Clearfield™, are not available.

These results support the hypothesis that roadside populations of canola in the U.S. are likely persistent from year to year, are capable of hybridizing to produce novel genotypes, and that escaped populations can contribute to the spread of transgenes outside of cultivation. Reports in Canada of feral populations of GE canola emerged soon after its commercial release there. Confirmation of GE pollen and crop movement among fields in Australia, U.K., Germany and France and Japan followed shortly thereafter. Ours is the first report of feral canola in the U.S. more than a decade after its commercial release. This delay raises questions of whether adequate oversight and monitoring protocols are in place in the U.S. to track the environmental impact of biotech products. At issue is the need to re-evaluate previous assumptions about crop systems: that crop genotypes outside of agriculture are not competitive; that protocols designed to reduce or prevent escape and proliferation of feral transgenic crops are effective; and that current tracking and monitoring of GE organisms are sufficient. Emerging pressures on agricultural systems by the accelerating growth of human populations argues that we take full advantage of the tools that biotechnology and conventional varietal development make available. It is essential that researchers, regulatory agencies and industry cooperate to ensure the continued security of food systems worldwide. The challenges of feeding a burgeoning global population in the face of limited and eroding natural resources requires substantial investments by all stakeholders. We must safely engage all tools available to us to advance food, fuel and fiber alternatives as modern agriculture rises to the challenges of the next decades.

## Supporting Information

Table S1 Supplemental table of all collected *B. napus* populations.
(DOCX)
