# Supplementary material for: The Establishment of Genetically Engineered Canola Populations in the U.S
Source: PLoS One. 2011 Oct 5;6(10):e25736. doi: 10.1371/journal.pone.0025736 (PMC3187797; doi:10.1371/journal.pone.0025736)
Supplement: Table S1 — Supplemental table of all collected B. napus populations. (DOCX) [file pone.0025736.s001.docx]

Table S1. Supplemental table of all collected B. napus populations. Populations where more than a single plant (9) was surveyed are highlighted in bold.

| Sample ID | Latitude | Longitude | Transgene State | Collector | Plot Size (m) | Plants per m^2^ | Mixed Populations |
| --- | --- | --- | --- | --- | --- | --- | --- |
| 1 | 46.09941667 | -96.83585 | LL+ | JPL | 100 | 0.02 |  |
| 2 | 46.05145 | -96.7284 | None | JPL | 100 | 0 |  |
| 3 | 46.05096667 | -96.62163333 | None | JPL | 100 | 0 |  |
| 4 | 46.05166667 | -96.84316667 | None | JPL | 100 | 0 |  |
| 5 | 46.07473333 | -96.93611667 | None | JPL | 100 | 0 |  |
| 6 | 46.06678333 | -97.03533333 | None | JPL | 100 | 0 |  |
| 7 | 46.06783333 | -97.14123333 | None | JPL | 100 | 0 |  |
| 8 | 46.07885 | -97.23261667 | None | JPL | 100 | 0 |  |
| 9 | 46.07963333 | -97.33555 | None | JPL | 100 | 0 |  |
| 10 | 46.07985 | -97.43941667 | None | JPL | 100 | 0 |  |
| 11 | 46.08035 | -97.53986667 | None | JPL | 100 | 0 |  |
| **12** | **46.08808333** | **-97.63348333** | **RR +** | **JPL** | **100** | **0.05** | **LL+; RR+** |
| 13 | 46.10265 | -97.73343333 | None | JPL | 100 | 0 |  |
| 14 | 46.10263333 | -97.83655 | None | JPL | 100 | 0 |  |
| 15 | 46.10983333 | -97.9378 | None | JPL | 100 | 0 |  |
| 16 | 46.1098 | -98.04278333 | None | JPL | 100 | 0 |  |
| 17 | 46.06448333 | -98.09111667 | None | JPL | 100 | 0 |  |
| 18 | 46.00851667 | -98.11666667 | None | JPL | 100 | 0 |  |
| 19 | 46.00858333 | -98.22103333 | None | JPL | 100 | 0 |  |
| 20 | 46.0086 | -98.32475 | None | JPL | 100 | 0 |  |
| 21 | 46.009 | -98.4284 | None | JPL | 100 | 0 |  |
| 22 | 46.00276667 | -98.54061667 | None | JPL | 100 | 0 |  |
| 23 | 46.00363333 | -98.64708333 | None | JPL | 100 | 0 |  |
| 24 | 46.02576667 | -98.7227 | None | JPL | 100 | 0 |  |
| 25 | 46.02563333 | -98.82533333 | None | JPL | 100 | 0 |  |
| 26 | 46.02683333 | -98.92853333 | None | JPL | 100 | 0 |  |
| 28 | 46.02678333 | -99.02925 | None | JPL | 100 | 0 |  |
| 29 | 46.02701667 | -99.13093333 | None | JPL | 100 | 0 |  |
| 30 | 46.027 | -99.23351667 | None | JPL | 100 | 0 |  |
| 31 | 46.03406667 | -99.33703333 | None | JPL | 100 | 0 |  |
| 32 | 46.0346 | -99.43366667 | None | JPL | 100 | 0 |  |
| 33 | 46.02761667 | -99.53468333 | None | JPL | 100 | 0 |  |
| 34 | 46.02801667 | -99.63726667 | None | JPL | 100 | 0 |  |
| 35 | 46.0278 | -99.74225 | None | JPL | 100 | 0 |  |
| 36 | 46.0136 | -99.82831667 | None | JPL | 100 | 0 |  |
| 37 | 46.02866667 | -99.91496667 | None | JPL | 100 | 0 |  |
| 38 | 46.02791667 | -100.0188667 | None | JPL | 100 | 0 |  |
| 39 | 46.09205 | -100.10375 | None | JPL | 100 | 0 |  |
| 40 | 46.14328333 | -100.1705167 | None | JPL | 100 | 0 |  |
| 41 | 46.20766667 | -100.2108167 | None | JPL | 100 | 0 |  |
| 42 | 46.25508333 | -100.2153333 | None | JPL | 100 | 0 |  |
| 43 | 46.24663333 | -100.1105 | None | JPL | 100 | 0 |  |
| 44 | 46.24626667 | -100.0078 | None | JPL | 100 | 0 |  |
| 45 | 46.26008333 | -99.90818333 | None | JPL | 100 | 0 |  |
| 46 | 46.25935 | -99.80675 | None | JPL | 100 | 0 |  |
| 47 | 46.25963333 | -99.70675 | None | JPL | 100 | 0 |  |
| 48 | 46.25928333 | -99.6029 | None | JPL | 100 | 0 |  |
| 49 | 46.25891667 | -99.49868333 | None | JPL | 100 | 0 |  |
| 50 | 46.26723333 | -99.39975 | None | JPL | 100 | 0 |  |
| 51 | 46.28333333 | -99.3252 | Null | JPL | 100 | 0.02 |  |
| 52 | 46.28311667 | -99.22481667 | None | JPL | 100 | 0 |  |
| 53 | 46.30125 | -99.1317 | None | JPL | 100 | 0 |  |
| 54 | 46.312 | -99.03053333 | None | JPL | 100 | 0 |  |
| 55 | 46.3313 | -98.95396667 | None | JPL | 100 | 0 |  |
| 56 | 46.35533333 | -98.88173333 | None | JPL | 100 | 0 |  |
| 57 | 46.35525 | -98.77848333 | None | JPL | 100 | 0 |  |
| 58 | 46.3553 | -98.67361667 | None | JPL | 100 | 0 |  |
| 59 | 46.3553 | -98.56993333 | None | JPL | 100 | 0 |  |
| 60 | 46.3555 | -98.46546667 | None | JPL | 100 | 0 |  |
| 61 | 46.35546667 | -98.35918333 | None | JPL | 100 | 0 |  |
| 62 | 46.35525 | -98.25205 | None | JPL | 100 | 0 |  |
| 63 | 46.35578333 | -98.15228333 | None | JPL | 100 | 0 |  |
| 64 | 46.38768333 | -98.07638333 | None | JPL | 100 | 0 |  |
| 65 | 46.44245 | -98.05398333 | None | JPL | 100 | 0 |  |
| 66 | 46.44208333 | -97.95951667 | None | JPL | 100 | 0 |  |
| 67 | 46.4419 | -97.85505 | None | JPL | 100 | 0 |  |
| 68 | 46.44208333 | -97.74965 | None | JPL | 100 | 0 |  |
| 69 | 46.44201667 | -97.64611667 | None | JPL | 100 | 0 |  |
| 70 | 46.44225 | -97.54101667 | None | JPL | 100 | 0 |  |
| 71 | 46.4793 | -97.48966667 | None | JPL | 100 | 0 |  |
| 72 | 46.5528 | -97.48981667 | None | JPL | 100 | 0 |  |
| 73 | 46.62473333 | -97.49038333 | RR + | JPL | 100 | 1.07 |  |
| 74 | 46.62978333 | -97.58975 | RR + | JPL | 100 | 0.02 |  |
| 75 | 46.62743333 | -97.60355 | RR + | JPL | 100 | 1 |  |
| 76 | 46.63001667 | -97.68748333 | LL+ | JPL | 100 | 2.2 |  |
| 77 | 46.63025 | -97.78975 | RR + | JPL | 100 | 2.36 |  |
| 78 | 46.87758333 | -97.02118333 | None | JPL | 50 | 0 |  |
| 79 | 46.8769 | -97.10516667 | RR + | JPL | 50 | 0.06 |  |
| 80 | 46.87413333 | -97.22273333 | RR + | JPL | 50 | 0.04 |  |
| 81 | 46.87583333 | -97.3494 | None | JPL | 50 | 0 |  |
| 82 | 46.87586667 | -97.44601667 | Null | JPL | 50 | 0.04 |  |
| 83 | 46.876 | -97.57318333 | RR + | JPL | 50 | 0.06 |  |
| 84 | 46.90798333 | -97.66588333 | RR + | JPL | 50 | 0.04 |  |
| 85 | 46.9202 | -97.80518333 | RR + | JPL | 50 | 0.06 |  |
| 86 | 46.92061667 | -97.92621667 | LL+ | JPL | 50 | 0.02 |  |
| 87 | 46.91206667 | -98.03355 | LL+ | JPL | 50 | 0.54 |  |
| 88 | 46.87208333 | -98.08085 | None | JPL | 100 | 0 |  |
| 89 | 46.79923333 | -98.08113333 | None | JPL | 100 | 0 |  |
| 90 | 46.7268 | -98.08161667 | None | JPL | 100 | 0 |  |
| 91 | 46.65661667 | -98.08166667 | LL+ | JPL | 100 | 0.15 |  |
| **92** | **46.63065** | **-97.97166667** | **LL+ ; RR+** | **JPL** | **100** | **0.09** | **LL+; RR+; LLRR+; Null** |
| 93 | 46.63025 | -97.87115 | None | JPL | 100 | 0 |  |
| 94 | 46.63101667 | -98.18663333 | LL+ | JPL | 100 | 0.01 |  |
| 95 | 46.63105 | -98.29088333 | RR + | JPL | 100 | 0.01 |  |
| 96 | 46.6311 | -98.39693333 | None | JPL | 100 | 0 |  |
| 97 | 46.6311 | -98.50226667 | None | JPL | 100 | 0 |  |
| 98 | 46.63098333 | -98.60775 | RR + | JPL | 100 | 0.04 |  |
| 99 | 46.63095 | -98.70826667 | LL+ | JPL | 100 | 0.09 |  |
| 100 | 46.63068333 | -98.81908333 | Null | JPL | 100 | 0.01 |  |
| 101 | 46.63055 | -98.92278333 | None | JPL | 100 | 0 |  |
| 102 | 46.63063333 | -99.03743333 | None | JPL | 100 | 0 |  |
| 103 | 46.63075 | -99.13145 | Null | JPL | 100 | 0.02 |  |
| 104 | 46.56735 | -99.1417 | None | JPL | 100 | 0 |  |
| 105 | 46.5294 | -99.19145 | None | JPL | 100 | 0 |  |
| 106 | 46.52925 | -99.29726667 | None | JPL | 100 | 0 |  |
| 107 | 46.51468333 | -99.38258333 | None | JPL | 100 | 0 |  |
| 108 | 46.51495 | -99.48798333 | None | JPL | 100 | 0 |  |
| 109 | 46.51488333 | -99.59458333 | None | JPL | 100 | 0 |  |
| 110 | 46.51528333 | -99.69326667 | None | JPL | 100 | 0 |  |
| 111 | 46.51258333 | -99.79863333 | None | JPL | 100 | 0 |  |
| 112 | 46.5014 | -99.89501667 | RR + | JPL | 100 | 0.05 |  |
| 113 | 46.48701667 | -99.98705 | None | JPL | 100 | 0 |  |
| 114 | 46.48728333 | -100.0895 | None | JPL | 100 | 0 |  |
| 115 | 46.48758333 | -100.1962 | None | JPL | 100 | 0 |  |
| 116 | 46.49025 | -100.2930833 | None | JPL | 100 | 0 |  |
| 117 | 46.57376667 | -100.29355 | None | JPL | 100 | 0 |  |
| 118 | 46.64741667 | -100.2911667 | None | JPL | 100 | 0 |  |
| **119** | **46.69878333** | **-100.2907333** | **LL+** | **JPL** | **100** | **0.11** | **LL+; RR+** |
| 120 | 46.777 | -100.2894667 | None | JPL | 100 | 0 |  |
| 121 | 46.86458333 | -100.1811833 | None | JPL | 50 | 0 |  |
| 122 | 46.86445 | -100.0700333 | None | JPL | 50 | 0 |  |
| 123 | 46.8645 | -99.95753333 | None | JPL | 50 | 0 |  |
| 124 | 46.87188333 | -99.85503333 | None | JPL | 50 | 0 |  |
| 125 | 46.87835 | -99.74728333 | None | JPL | 50 | 0 |  |
| 126 | 46.87976667 | -99.63786667 | None | JPL | 50 | 0 |  |
| 127 | 46.87991667 | -99.49195 | None | JPL | 50 | 0 |  |
| 128 | 46.87791667 | -99.38261667 | None | JPL | 50 | 0 |  |
| 129 | 46.87863333 | -99.27208333 | None | JPL | 50 | 0 |  |
| 130 | 46.88661667 | -99.16108333 | None | JPL | 50 | 0 |  |
| 131 | 46.8911 | -99.06933333 | None | JPL | 50 | 0 |  |
| 132 | 46.89211667 | -98.94345 | None | JPL | 50 | 0 |  |
| 133 | 46.89226667 | -98.81111667 | None | JPL | 50 | 0 |  |
| 134 | 46.89213333 | -98.67903333 | LL+ | JPL | 50 | 0.02 |  |
| 135 | 46.97705 | -98.74975 | None | JPL | 100 | 0 |  |
| 136 | 47.04135 | -98.80726667 | RR + | JPL | 100 | 0.07 |  |
| 137 | 47.10205 | -98.85643333 | Null | JPL | 100 | 0.02 |  |
| 138 | 47.16606667 | -98.9059 | LL+ | JPL | 100 | 0.1 |  |
| 139 | 47.16796667 | -99.01268333 | None | JPL | 100 | 0 |  |
| 140 | 47.16566667 | -99.11743333 | None | JPL | 100 | 0 |  |
| 141 | 47.15353333 | -99.21375 | None | JPL | 100 | 0 |  |
| 142 | 47.1538 | -99.30131667 | None | JPL | 100 | 0 |  |
| 143 | 47.1394 | -99.41046667 | None | JPL | 100 | 0 |  |
| 144 | 47.13983333 | -99.51383333 | None | JPL | 100 | 0 |  |
| 145 | 47.14011667 | -99.72308333 | None | JPL | 100 | 0 |  |
| 146 | 47.14011667 | -99.72305 | None | JPL | 100 | 0 |  |
| 147 | 47.1417 | -99.82973333 | None | JPL | 100 | 0 |  |
| 148 | 47.14006667 | -99.93415 | None | JPL | 100 | 0 |  |
| 149 | 47.14023333 | -100.03735 | None | JPL | 100 | 0 |  |
| 150 | 47.14121667 | -100.1441333 | None | JPL | 100 | 0 |  |
| 151 | 47.14133333 | -100.2469833 | None | JPL | 100 | 0 |  |
| 152 | 47.14196667 | -100.3537333 | None | JPL | 100 | 0 |  |
| 153 | 47.14223333 | -100.4617333 | None | JPL | 100 | 0 |  |
| 154 | 47.14228333 | -100.5660333 | None | JPL | 100 | 0 |  |
| 155 | 47.14265 | -100.6719333 | None | JPL | 100 | 0 |  |
| 156 | 47.14261667 | -100.7782333 | None | JPL | 100 | 0 |  |
| 157 | 47.06671667 | -100.7921167 | None | JPL | 100 | 0 |  |
| 158 | 46.99483333 | -100.79205 | None | JPL | 100 | 0 |  |
| 159 | 46.92363333 | -100.77195 | None | JPL | 100 | 0 |  |
| 160 | 46.82698333 | -100.885 | LL+ | JPL | 50 | 0.02 |  |
| 161 | 46.75241667 | -100.9015667 | RR + | JPL | 100 | 0.01 |  |
| 162 | 46.6796 | -100.9025333 | None | JPL | 100 | 0 |  |
| 163 | 46.60833333 | -100.9031167 | None | JPL | 100 | 0 |  |
| 164 | 46.53525 | -100.9031333 | None | JPL | 100 | 0 |  |
| 165 | 46.47188333 | -100.9209167 | None | JPL | 100 | 0 |  |
| 166 | 46.47286667 | -101.0266167 | None | JPL | 100 | 0 |  |
| 167 | 46.45475 | -101.1198667 | None | JPL | 100 | 0 |  |
| 168 | 46.45843333 | -101.2382167 | None | JPL | 100 | 0 |  |
| 169 | 46.4439 | -101.3328833 | None | JPL | 100 | 0 |  |
| 170 | 46.43193333 | -101.4319833 | None | JPL | 100 | 0 |  |
| 171 | 46.41501667 | -101.5318333 | None | JPL | 100 | 0 |  |
| 172 | 46.40065 | -101.6177833 | None | JPL | 100 | 0 |  |
| 173 | 46.40093333 | -101.7300833 | None | JPL | 100 | 0 |  |
| 174 | 46.40135 | -101.82755 | None | JPL | 100 | 0 |  |
| 175 | 46.38316667 | -101.9244667 | Null | JPL | 100 | 0.01 |  |
| 176 | 46.33186667 | -101.96495 | LL+ | JPL | 100 | 0.01 |  |
| **177** | **46.26086667** | **-101.9572667** | **RR +** | **JPL** | **100** | **0.06** | **LL+; RR+** |
| 178 | 46.37163333 | -102.0678 | RR + | JPL | 100 | 0.01 |  |
| 179 | 46.3703 | -102.1733167 | None | JPL | 100 | 0 |  |
| 180 | 46.3715 | -102.278 | None | JPL | 100 | 0 |  |
| 181 | 46.37813333 | -102.3179167 | RR + | JPL | 100 | 0.25 |  |
| 182 | 46.4526 | -102.31815 | None | JPL | 100 | 0 |  |
| 183 | 46.52346667 | -102.3181833 | None | JPL | 100 | 0 |  |
| 184 | 46.36368333 | -102.3981833 | None | JPL | 100 | 0 |  |
| 185 | 46.34633333 | -102.46475 | None | JPL | 100 | 0 |  |
| 186 | 46.27555 | -102.4769667 | None | JPL | 100 | 0 |  |
| 187 | 46.39183333 | -102.50655 | None | JPL | 100 | 0 |  |
| 188 | 46.41318333 | -102.6001833 | Null | JPL | 100 | 0.03 |  |
| 189 | 46.41288333 | -102.6967167 | None | JPL | 100 | 0 |  |
| 190 | 46.41215 | -102.8120667 | None | JPL | 100 | 0 |  |
| 191 | 46.41215 | -102.8627167 | RR + | JPL | 100 | 0.04 |  |
| 192 | 46.33943333 | -102.8625333 | None | JPL | 100 | 0 |  |
| 193 | 46.26555 | -102.8672167 | None | JPL | 100 | 0 |  |
| 194 | 46.48216667 | -102.86375 | None | JPL | 100 | 0 |  |
| 195 | 46.52871667 | -102.86665 | RR + | JPL | 100 | 0.01 |  |
| 196 | 46.52621667 | -102.9773667 | RR + | JPL | 100 | 0.01 |  |
| 197 | 46.52623333 | -103.08125 | RR + | JPL | 100 | 0.01 |  |
| 198 | 46.52603333 | -103.1954 | None | JPL | 100 | 0 |  |
| 199 | 46.48255 | -103.2543333 | None | JPL | 100 | 0 |  |
| 200 | 46.48255 | -103.32725 | None | JPL | 100 | 0 |  |
| 201 | 46.6014 | -103.19585 | None | JPL | 100 | 0 |  |
| 202 | 46.67486667 | -103.1897833 | None | JPL | 100 | 0 |  |
| 203 | 46.7469 | -103.1893167 | None | JPL | 100 | 0 |  |
| 204 | 46.81916667 | -103.1900833 | None | JPL | 100 | 0 |  |
| 205 | 46.88903333 | -103.1896333 | LL+ | JPL | 50 | 0.02 |  |
| 206 | 46.89108333 | -103.0934 | None | JPL | 50 | 0 |  |
| 207 | 46.88708333 | -102.9987167 | None | JPL | 50 | 0 |  |
| 208 | 46.89045 | -102.8863 | None | JPL | 50 | 0 |  |
| 209 | 46.89588333 | -102.7819 | None | JPL | 50 | 0 |  |
| 210 | 46.88323333 | -102.7056667 | RR + | JPL | 50 | 0.2 |  |
| 211 | 46.88131667 | -102.5851667 | RR + | JPL | 50 | 0.44 |  |
| 212 | 46.87675 | -102.4733833 | RR + | JPL | 50 | 2 |  |
| 213 | 46.87705 | -102.3937333 | RR + | JPL | 50 | 0.5 |  |
| **215** | **46.85298333** | **-102.2061333** | **RR +** | **JPL** | **50** | **20** | **RR+; Null** |
| 216 | 46.86258333 | -102.0583 | RR + | JPL | 50 | 20 |  |
| 217 | 46.863 | -101.9771 | RR + | JPL | 50 | 0.2 |  |
| 218 | 46.8617 | -101.8364 | RR + | JPL | 50 | 0.5 |  |
| 219 | 46.86156667 | -101.7185333 | Null | JPL | 50 | 0.1 |  |
| 220 | 46.86148333 | -101.58935 | None | JPL | 50 | 0 |  |
| 221 | 46.86123333 | -101.4786 | RR + | JPL | 50 | 0.2 |  |
| 222 | 46.86093333 | -101.3514333 | None | JPL | 50 | 0 |  |
| 223 | 46.86203333 | -101.2467167 | RR + | JPL | 50 | 0.04 |  |
| 224 | 46.8621 | -101.1384833 | None | JPL | 50 | 0 |  |
| 225 | 46.86268333 | -101.0313167 | None | JPL | 50 | 0 |  |
| 226 | 46.84983333 | -100.92 | RR + | JPL | 50 | 0.02 |  |
| 227 | 46.83043333 | -100.8070167 | None | JPL | 50 | 0 |  |
| 228 | 47.18736667 | -100.8546 | None | JPL | 100 | 0 |  |
| 229 | 47.22546667 | -100.9341667 | None | JPL | 100 | 0 |  |
| 230 | 47.2779 | -101.0046167 | None | JPL | 100 | 0 |  |
| 231 | 47.30301667 | -101.0357667 | RR + | JPL | 100 | 0.06 |  |
| 232 | 47.26401667 | -101.11595 | RR + | JPL | 100 | 0.01 |  |
| 233 | 47.2371 | -101.2102167 | LL+ | JPL | 100 | 0.03 |  |
| 234 | 47.25691667 | -101.3002833 | RR + | JPL | 100 | 0.01 |  |
| 235 | 47.29473333 | -101.3811833 | None | JPL | 100 | 0 |  |
| 236 | 47.29926667 | -101.4884333 | RR + | JPL | 100 | 0.01 |  |
| 237 | 47.29936667 | -101.5875833 | RR + | JPL | 100 | 0.77 |  |
| 238 | 47.29921667 | -101.68605 | LL+ | JPL | 100 | 0.04 |  |
| 239 | 47.30276667 | -101.7855833 | LL+ | JPL | 100 | 0.08 |  |
| 240 | 47.29211667 | -101.8868667 | None | JPL | 100 | 0 |  |
| 241 | 47.30146667 | -101.9812833 | None | JPL | 100 | 0 |  |
| 242 | 47.2992 | -102.07305 | None | JPL | 100 | 0 |  |
| 243 | 47.29961667 | -102.1762333 | None | JPL | 100 | 0 |  |
| 244 | 47.31376667 | -102.2743 | Null | JPL | 100 | 0.02 |  |
| 245 | 47.31511667 | -102.3336167 | RR + | JPL | 100 | 0.06 |  |
| 246 | 47.34276667 | -102.4154333 | None | JPL | 100 | 0 |  |
| 247 | 47.34366667 | -102.5135667 | None | JPL | 100 | 0 |  |
| 248 | 47.34958333 | -102.6158 | RR + | JPL | 100 | 0.01 |  |
| 249 | 47.3575 | -102.7056167 | None | JPL | 100 | 0 |  |
| 250 | 47.35768333 | -102.8163667 | None | JPL | 100 | 0 |  |
| 251 | 47.35773333 | -102.92285 | None | JPL | 100 | 0 |  |
| 252 | 47.35738333 | -103.0310667 | None | JPL | 100 | 0 |  |
| 253 | 47.34325 | -103.1275 | None | JPL | 100 | 0 |  |
| 254 | 47.36073333 | -103.2015833 | None | JPL | 100 | 0 |  |
| 255 | 47.42721667 | -103.2481333 | None | JPL | 100 | 0 |  |
| 256 | 47.49865 | -103.24815 | None | JPL | 100 | 0 |  |
| 257 | 47.57078333 | -103.23775 | None | JPL | 100 | 0 |  |
| 258 | 47.62825 | -103.2695167 | None | JPL | 100 | 0 |  |
| 259 | 47.69925 | -103.2831833 | None | JPL | 100 | 0 |  |
| 260 | 47.7557 | -103.2830667 | None | JPL | 100 | 0 |  |
| 261 | 47.79903333 | -103.3135333 | None | JPL | 100 | 0 |  |
| 262 | 47.80488333 | -103.41975 | None | JPL | 100 | 0 |  |
| 263 | 47.8461 | -103.64235 | None | JPL | 100 | 0 |  |
| 264 | 47.86271667 | -104.00005 | None | JPL | 100 | 0 |  |
| 265 | 48.13998333 | -103.7222667 | None | JPL | 100 | 0 |  |
| 266 | 48.14716667 | -103.6016333 | LL+ | JPL | 100 | 0.01 |  |
| 267 | 48.15438333 | -103.4853 | None | JPL | 100 | 0 |  |
| 268 | 48.15443333 | -103.38135 | None | JPL | 100 | 0 |  |
| 269 | 48.15671667 | -103.27525 | None | JPL | 100 | 0 |  |
| 270 | 48.17848333 | -103.1884167 | None | JPL | 100 | 0 |  |
| 271 | 48.1817 | -103.10875 | None | JPL | 100 | 0 |  |
| 272 | 48.1974 | -103.0232667 | None | JPL | 100 | 0 |  |
| 273 | 48.19721667 | -102.91115 | None | JPL | 100 | 0 |  |
| 274 | 48.20685 | -102.81335 | None | JPL | 100 | 0 |  |
| 275 | 48.21145 | -102.7090833 | None | JPL | 100 | 0 |  |
| 276 | 48.20393333 | -102.6126 | None | JPL | 100 | 0 |  |
| 277 | 48.13183333 | -102.6126333 | None | JPL | 100 | 0 |  |
| 278 | 48.05968333 | -102.60855 | None | JPL | 100 | 0 |  |
| 279 | 48.0347 | -102.5276667 | None | JPL | 100 | 0 |  |
| 280 | 47.97998333 | -102.4592167 | None | JPL | 100 | 0 |  |
| 281 | 47.97775 | -102.34095 | None | JPL | 100 | 0 |  |
| 282 | 47.97773333 | -102.2361667 | RR + | JPL | 100 | 0.1 |  |
| 283 | 47.97783333 | -102.1277 | RR + | JPL | 100 | 1 |  |
| 284 | 47.97783333 | -102.0217667 | RR + | JPL | 100 | 0.85 |  |
| 285 | 47.97818333 | -101.9146833 | Null | JPL | 100 | 0.37 |  |
| 286 | 47.97786667 | -101.8147167 | RR + | JPL | 100 | 0.38 |  |
| 287 | 47.97806667 | -101.7075833 | RR + | JPL | 100 | 0.2 |  |
| 288 | 47.97816667 | -101.60455 | RR + | JPL | 100 | 0.07 |  |
| 289 | 47.9787 | -101.5036167 | RR + | JPL | 100 | 0.11 |  |
| 290 | 47.97865 | -101.4006167 | RR + | JPL | 100 | 0.02 |  |
| 291 | 47.97881667 | -101.29185 | RR + | JPL | 100 | 0.04 |  |
| 292 | 47.97886667 | -101.1915333 | LL+ | JPL | 100 | 0.15 |  |
| **293** | **47.97926667** | **-101.0896** | **LL+** | **JPL** | **100** | **2.08** | **LL+; RR+; Null** |
| 294 | 47.99306667 | -100.98415 | RR + | JPL | 100 | 2 |  |
| 295 | 48.02341667 | -100.9293667 | RR + | JPL | 100 | 10 |  |
| **296** | **48.0553** | **-100.9310833** | **RR +** | **JPL** | **100** | **2** | **LL+; RR+** |
| 297 | 48.0205 | -100.8564333 | RR + | JPL | 100 | 2 |  |
| 298 | 48.00623333 | -100.73615 | RR + | JPL | 100 | 0.16 |  |
| 299 | 47.97823333 | -100.6335333 | RR + | JPL | 100 | 0.16 |  |
| 300 | 47.9502 | -100.5379 | RR + | JPL | 100 | 0.02 |  |
| 301 | 47.8883 | -100.5209833 | None | JPL | 100 | 0 |  |
| 302 | 47.83325 | -100.5493833 | None | JPL | 100 | 0 |  |
| 303 | 47.83345 | -100.6499333 | None | JPL | 100 | 0 |  |
| 304 | 47.82658333 | -100.75995 | None | JPL | 100 | 0 |  |
| 305 | 47.8339 | -100.85875 | None | JPL | 100 | 0 |  |
| 306 | 47.81925 | -100.96145 | Null | JPL | 100 | 0.18 |  |
| 307 | 47.8194 | -101.0661167 | None | JPL | 100 | 0 |  |
| 308 | 47.81928333 | -101.16225 | Null | JPL | 100 | 0.02 |  |
| 309 | 47.81935 | -101.2677167 | LL+ | JPL | 100 | 0.02 |  |
| 310 | 47.83383333 | -101.2927667 | LL+ | JPL | 100 | 0.17 |  |
| 311 | 47.9226 | -101.2911167 | RR + | JPL | 100 | 0.06 |  |
| 312 | 48.05598333 | -101.2956167 | None | JPL | 100 | 0 |  |
| 313 | 47.80931667 | -101.2932667 | RR + | JPL | 100 | 0.75 |  |
| 314 | 47.74656667 | -101.2934833 | LL+ | JPL | 100 | 0.43 |  |
| 315 | 47.67411667 | -101.2893 | LL+ | JPL | 100 | 1.07 |  |
| 316 | 47.64693333 | -101.36955 | LL+ | JPL | 100 | 0.01 |  |
| 317 | 47.63943333 | -101.4740333 | None | JPL | 100 | 0 |  |
| 318 | 47.64656667 | -101.5786833 | None | JPL | 100 | 0 |  |
| 319 | 47.64648333 | -101.68565 | None | JPL | 100 | 0 |  |
| 320 | 47.64651667 | -101.7910833 | LL+ | JPL | 100 | 0.01 |  |
| 321 | 47.57443333 | -101.2604833 | RR + | JPL | 100 | 0.06 |  |
| 322 | 47.51623333 | -101.1849333 | RR + | JPL | 100 | 0.02 |  |
| 323 | 47.50205 | -101.2865833 | None | JPL | 100 | 0 |  |
| 324 | 47.4583 | -101.1237333 | RR + | JPL | 100 | 0.11 |  |
| 325 | 47.45838333 | -101.005 | LL+ | JPL | 100 | 0.01 |  |
| 326 | 47.46471667 | -100.9077167 | LL+ | JPL | 100 | 0.01 |  |
| 327 | 47.48716667 | -100.8369333 | None | JPL | 100 | 0 |  |
| 328 | 47.48721667 | -100.7318 | None | JPL | 100 | 0 |  |
| 329 | 47.46915 | -100.6302333 | RR + | JPL | 100 | 0.01 |  |
| 330 | 47.47661667 | -100.5265833 | LL+ | JPL | 100 | 0.07 |  |
| 331 | 47.48726667 | -100.4126667 | Null | JPL | 100 | 0.01 |  |
| 332 | 47.48695 | -100.3052 | RR + | JPL | 100 | 0.08 |  |
| 333 | 47.48566667 | -100.19 | RR + | JPL | 100 | 0.03 |  |
| 334 | 47.46031667 | -100.1185667 | LL+ | JPL | 100 | 0.02 |  |
| 335 | 47.44323333 | -100.0327833 | None | JPL | 100 | 0 |  |
| 336 | 47.44316667 | -99.92625 | RR + | JPL | 100 | 0.44 |  |
| 337 | 47.44311667 | -99.8196 | None | JPL | 100 | 0 |  |
| 338 | 47.45765 | -99.72215 | None | JPL | 100 | 0 |  |
| 339 | 47.4577 | -99.61861667 | Null | JPL | 100 | 0.07 |  |
| 340 | 47.45756667 | -99.51155 | RR + | JPL | 100 | 0.01 |  |
| 341 | 47.45736667 | -99.40215 | Null | JPL | 100 | 0.05 |  |
| 342 | 47.45763333 | -99.29301667 | LL+ | JPL | 100 | 0.18 |  |
| 343 | 47.45748333 | -99.19521667 | Null | JPL | 100 | 0.08 |  |
| 344 | 47.45645 | -99.13188333 | RR + | JPL | 100 | 0.1 |  |
| 345 | 47.45706667 | -98.9947 | RR + | JPL | 100 | 0.02 |  |
| 346 | 47.457 | -98.88338333 | LL+ | JPL | 100 | 0.03 |  |
| 347 | 47.45703333 | -98.78045 | Null | JPL | 100 | 0.02 |  |
| 348 | 47.45715 | -98.67668333 | None | JPL | 100 | 0 |  |
| 349 | 47.45686667 | -98.57573333 | LL+ | JPL | 100 | 0.02 |  |
| 350 | 47.44236667 | -98.4738 | None | JPL | 100 | 0 |  |
| 351 | 47.4424 | -98.33573333 | None | JPL | 100 | 0 |  |
| 352 | 47.44236667 | -98.2135 | None | JPL | 100 | 0 |  |
| 353 | 47.44188333 | -98.1093 | RR + | JPL | 100 | 0.05 |  |
| 354 | 47.43846667 | -97.99585 | None | JPL | 100 | 0 |  |
| 355 | 47.44173333 | -97.88316667 | None | JPL | 100 | 0 |  |
| 356 | 47.47805 | -97.83601667 | RR + | JPL | 100 | 0.01 |  |
| 357 | 47.51393333 | -97.78286667 | RR + | JPL | 100 | 0.02 |  |
| 358 | 47.5207 | -97.68063333 | None | JPL | 100 | 0 |  |
| 359 | 47.52745 | -97.57573333 | None | JPL | 100 | 0 |  |
| 360 | 47.52715 | -97.4839 | None | JPL | 100 | 0 |  |
| 361 | 47.49828333 | -97.39393333 | None | JPL | 100 | 0 |  |
| 362 | 47.49786667 | -97.26976667 | LL+ | JPL | 100 | 0.04 |  |
| 363 | 47.49775 | -97.18266667 | None | JPL | 100 | 0 |  |
| 364 | 47.48013333 | -97.07671667 | Null | JPL | 50 | 0.2 |  |
| 365 | 47.40011667 | -97.074 | None | JPL | 50 | 0 |  |
| 366 | 47.35328333 | -97.05151667 | LL+ | JPL | 50 | 0.1 |  |
| 367 | 47.28183333 | -97.02023333 | RR + | JPL | 50 | 0.06 |  |
| 368 | 47.19353333 | -96.99025 | RR + | JPL | 50 | 0.1 |  |
| 369 | 47.11668333 | -96.95901667 | Null | JPL | 50 | 0.06 |  |
| 370 | 47.04993333 | -96.9307 | RR + | JPL | 50 | 0.16 |  |
| 372 | 46.63026667 | -96.83458333 | Null | JPL | 100 | 0.07 |  |
| 373 | 46.63023333 | -96.94321667 | LL+ | JPL | 100 | 0.24 |  |
| 374 | 46.62995 | -97.05568333 | Null | JPL | 100 | 0.05 |  |
| 375 | 46.62968333 | -97.16573333 | LL+ | JPL | 100 | 0.02 |  |
| **376** | **46.6294** | **-97.27573333** | **RR +** | **JPL** | **100** | **1.7** | **LL+; RR+; Null** |
| 377 | 46.62931667 | -97.3851 | RR + | JPL | 100 | 0.71 |  |
| 378 | 47.77345 | -97.11051667 | Null | MS | 100 | 0.05 |  |
| 379 | 47.77375 | -97.22016667 | None | MS | 100 | 0 |  |
| 380 | 47.7591 | -97.30331667 | RR + | MS | 100 | 0.01 |  |
| 381 | 47.7447 | -97.37755 | None | MS | 100 | 0 |  |
| 382 | 47.74443333 | -97.47411667 | LL+ | MS | 100 | 0.27 |  |
| 383 | 47.74445 | -97.56285 | LL+ | MS | 100 | 0.37 |  |
| 384 | 47.74476667 | -97.66023333 | Null | MS | 100 | 0.02 |  |
| 385 | 47.7447 | -97.76418333 | None | MS | 100 | 0 |  |
| 386 | 47.74476667 | -97.86073333 | LL+ ; RR+ | MS | 100 | 0.01 |  |
| 387 | 47.74471667 | -97.95781667 | None | MS | 100 | 0 |  |
| 388 | 47.74506667 | -98.05081667 | None | MS | 100 | 0 |  |
| 389 | 47.74936667 | -98.15061667 | LL+ | MS | 100 | 0.02 |  |
| 390 | 47.78826667 | -98.27938333 | None | MS | 100 | 0 |  |
| 391 | 47.80278333 | -98.37638333 | None | MS | 100 | 0 |  |
| 392 | 47.80283333 | -98.48308333 | None | MS | 100 | 0 |  |
| 393 | 47.80011667 | -98.58665 | None | MS | 100 | 0 |  |
| 394 | 47.7241 | -98.59005 | None | MS | 100 | 0 |  |
| 395 | 47.68701667 | -98.66061667 | None | MS | 100 | 0 |  |
| 396 | 47.68706667 | -98.74776667 | LL+ | MS | 100 | 0.02 |  |
| 397 | 47.68723333 | -98.8554 | None | MS | 100 | 0 |  |
| 398 | 47.68726667 | -98.96478333 | None | MS | 100 | 0 |  |
| 399 | 47.68735 | -99.07231667 | None | MS | 100 | 0 |  |
| 400 | 47.646 | -99.13261667 | None | MS | 100 | 0 |  |
| 401 | 47.64565 | -99.18103333 | LL+ | MS | 100 | 0.02 |  |
| 402 | 47.64558333 | -99.28573333 | None | MS | 100 | 0 |  |
| 403 | 47.64596667 | -99.37851667 | Null | MS | 100 | 0.05 |  |
| 404 | 47.64578333 | -99.47976667 | None | MS | 100 | 0 |  |
| 405 | 47.64625 | -99.58613333 | None | MS | 100 | 0 |  |
| 406 | 47.68593333 | -99.7263 | Null | MS | 100 | 0.03 |  |
| 407 | 47.71678333 | -99.80985 | None | MS | 100 | 0 |  |
| 408 | 47.746 | -99.89286667 | RR + | MS | 100 | 0.01 |  |
| 409 | 47.78706667 | -99.97808333 | LL+ | MS | 100 | 0.02 |  |
| 410 | 47.81595 | -100.0790667 | RR + | MS | 100 | 0.09 |  |
| 411 | 47.85376667 | -100.1769333 | RR + | MS | 100 | 0.02 |  |
| 412 | 47.89078333 | -100.2673333 | LL+ | MS | 100 | 0.03 |  |
| 413 | 47.92018333 | -100.3771833 | Null | MS | 100 | 0.81 |  |
| 414 | 48.63076667 | -98.8083 | LL+ | MS | 100 | 0.09 |  |
| 415 | 48.63135 | -98.91555 | LL+ | MS | 100 | 0.28 |  |
| 416 | 48.63121667 | -98.03163333 | LL+ | MS | 100 | 0.02 |  |
| 417 | 48.63115 | -99.13376667 | LL+ | MS | 100 | 0.08 |  |
| 418 | 48.63148333 | -99.24225 | Null | MS | 100 | 0.91 |  |
| 419 | 48.63178333 | -99.35775 | Null | MS | 100 | 0.04 |  |
| 420 | 48.632 | -99.47148333 | LL+ | MS | 100 | 0.08 |  |
| 421 | 48.63198333 | -99.58056667 | Null | MS | 100 | 0.05 |  |
| 422 | 48.66096667 | -99.667 | LL+ | MS | 100 | 0.07 |  |
| 423 | 48.6609 | -99.77185 | RR + | MS | 100 | 0.15 |  |
| 424 | 48.67546667 | -99.87353333 | LL+ | MS | 100 | 0.02 |  |
| 425 | 48.67698333 | -99.97201667 | LL+ | MS | 100 | 0.06 |  |
| 426 | 48.63058333 | -98.69218333 | LL+ | MS | 100 | 0.34 |  |
| 427 | 48.63031667 | -98.57923333 | LL+ | MS | 100 | 0.08 |  |
| 428 | 48.6302 | -98.47053333 | LL+ | MS | 100 | 0.05 |  |
| 429 | 48.63051667 | -98.36641667 | LL+ | MS | 100 | 0.02 |  |
| 430 | 48.63035 | -98.27485 | LL+ | MS | 100 | 3.06 |  |
| 431 | 48.62995 | -98.16628333 | LL+ | MS | 100 | 0.16 |  |
| 432 | 48.63018333 | -98.06431667 | Null | MS | 100 | 0.05 |  |
| 433 | 48.62993333 | -98.97091667 | LL+ | MS | 100 | 0.06 |  |
| 434 | 48.62281667 | -97.86236667 | RR + | MS | 100 | 0.04 |  |
| 435 | 48.6008 | -97.79666667 | LL+ | MS | 100 | 0.17 |  |
| 436 | 48.60236667 | -97.69356667 | Null | MS | 100 | 0.04 |  |
| 437 | 48.60105 | -97.59603333 | None | MS | 100 | 0 |  |
| 438 | 48.6005 | -97.4908 | None | MS | 100 | 0 |  |
| 439 | 48.57171667 | -97.42563333 | Null | MS | 100 | 0.31 |  |
| 440 | 48.57165 | -97.32146667 | LL+ | MS | 100 | 0.02 |  |
| 441 | 48.57115 | -97.21225 | None | MS | 100 | 0 |  |
| 442 | 48.76101667 | -98.25573333 | None | MS | 100 | 0 |  |
| 443 | 48.7613 | -98.15733333 | LL+ | MS | 100 | 0.11 |  |
| 444 | 48.761 | -98.05396667 | LL+ | MS | 100 | 0.48 |  |
| 445 | 48.76101667 | -97.93455 | RR + | MS | 100 | 0.14 |  |
| 446 | 48.76075 | -97.84005 | Null | MS | 100 | 0.04 |  |
| 447 | 48.77308333 | -97.73401667 | LL+ | MS | 100 | 0.16 |  |
| 448 | 48.79341667 | -97.62813333 | None | MS | 100 | 0 |  |
| 449 | 48.80443333 | -97.52363333 | LL+ | MS | 100 | 0.05 |  |
| 450 | 48.804 | -97.4143 | LL+ | MS | 100 | 0.14 |  |
| 451 | 48.80323333 | -97.2939 | RR + | MS | 100 | 0.04 |  |
| 452 | 48.8038 | -97.21321667 | None | MS | 100 | 0 |  |
| 453 | 48.414 | -98.84021667 | None | MS | 100 | 0 |  |
| 454 | 48.41351667 | -98.73153333 | None | MS | 100 | 0 |  |
| 455 | 48.41305 | -98.62286667 | LL+ | MS | 100 | 0.02 |  |
| 456 | 48.41285 | -98.51408333 | None | MS | 100 | 0 |  |
| 457 | 48.41293333 | -98.40541667 | None | MS | 100 | 0 |  |
| 458 | 48.41306667 | -98.30256667 | LL+ | MS | 100 | 0.03 |  |
| 459 | 48.41271667 | -98.18791667 | LL+ | MS | 100 | 0.02 |  |
| 460 | 48.41241667 | -98.07923333 | LL+ | MS | 100 | 0.01 |  |
| 461 | 48.487 | -98.97765 | LL+ | MS | 100 | 0.01 |  |
| 462 | 48.39768333 | -97.86201667 | None | MS | 100 | 0 |  |
| 463 | 48.39855 | -97.75836667 | LL+ | MS | 100 | 0.07 |  |
| 464 | 48.39805 | -97.64805 | LL+ | MS | 100 | 0.08 |  |
| 465 | 48.41228333 | -97.55743333 | Null | MS | 100 | 0.1 |  |
| 466 | 48.41221667 | -97.44843333 | None | MS | 100 | 0 |  |
| 467 | 48.41233333 | -97.349 | None | MS | 100 | 0 |  |
| 468 | 48.41198333 | -97.24478333 | Null | MS | 100 | 0.09 |  |
| 469 | 48.45181667 | -98.88433333 | LL+ | MS | 100 | 0.21 |  |
| 470 | 48.48628333 | -98.92275 | None | MS | 100 | 0 |  |
| 471 | 48.412 | -98.01423333 | None | MS | 100 | 0 |  |
| 472 | 48.4865 | -99.11388333 | Null | MS | 100 | 0.04 |  |
| 473 | 48.48676667 | -99.20388333 | Null | MS | 100 | 0.02 |  |
| 474 | 48.48685 | -99.31913333 | Null | MS | 100 | 0.02 |  |
| 475 | 48.48693333 | -99.43698333 | Null | MS | 100 | 0.01 |  |
| 476 | 48.49518333 | -99.52638333 | None | MS | 100 | 0 |  |
| 477 | 48.5018 | -99.62368333 | None | MS | 100 | 0 |  |
| 478 | 48.5016 | -99.72858333 | RR + | MS | 100 | 0.12 |  |
| 479 | 48.50128333 | -99.81981667 | None | MS | 100 | 0 |  |
| 480 | 48.50261667 | -99.90646667 | RR + | MS | 100 | 0.08 |  |
| 481 | 48.50166667 | -99.98833333 | None | MS | 100 | 0 |  |
| 482 | 48.50175 | -100.0807833 | None | MS | 100 | 0 |  |
| 483 | 48.50401667 | -100.1745167 | None | MS | 100 | 0 |  |
| 484 | 48.55061667 | -100.2291667 | None | MS | 100 | 0 |  |
| 485 | 48.59216667 | -100.2767333 | None | MS | 100 | 0 |  |
| 486 | 48.619 | -100.299 | None | MS | 100 | 0 |  |
| 487 | 48.33861667 | -100.4027 | None | MS | 100 | 0 |  |
| 488 | 48.35458333 | -100.3457333 | None | MS | 100 | 0 |  |
| 489 | 48.35495 | -100.2740833 | LL+ | MS | 100 | 0.08 |  |
| 490 | 48.35468333 | -100.1717333 | Null | MS | 100 | 0.43 |  |
| 491 | 48.35476667 | -100.0686333 | Null | MS | 100 | 0.21 |  |
| 492 | 48.35445 | -99.9652 | Null | MS | 100 | 0.07 |  |
| 493 | 48.35388333 | -99.8691 | None | MS | 100 | 0 |  |
| 494 | 48.33976667 | -99.79011667 | None | MS | 100 | 0 |  |
| 495 | 48.33921667 | -99.69658333 | Null | MS | 100 | 0.02 |  |
| 496 | 48.32451667 | -99.62056667 | None | MS | 100 | 0 |  |
| 497 | 48.30701667 | -99.53738333 | RR + | MS | 100 | 0.07 |  |
| 498 | 48.29178333 | -99.45928333 | Null | MS | 100 | 0.26 |  |
| 499 | 48.28036667 | -99.35698333 | RR + | MS | 100 | 0.13 |  |
| 500 | 48.27653333 | -99.26518333 | LL+ | MS | 100 | 0.04 |  |
| 501 | 48.26016667 | -99.17911667 | None | MS | 100 | 0 |  |
| 502 | 48.2223 | -99.0992 | None | MS | 100 | 0 |  |
| 503 | 48.14603333 | -98.93288333 | None | MS | 100 | 0 |  |
| 504 | 48.1039 | -98.86855 | None | MS | 100 | 0 |  |
| 505 | 48.76125 | -98.3917 | LL+ | MS | 100 | 15 |  |
| 506 | 48.76156667 | -98.47473333 | RR + | MS | 100 | 20 |  |
| **507** | **48.76151667** | **-98.56241667** | **LL+** | **MS** | **100** | **30** | **LL+, RR+** |
| 508 | 48.76148333 | -98.64988333 | RR + | MS | 100 | 15 |  |
| 509 | 48.7615 | -98.73728333 | RR + | MS | 100 | 10 |  |
| 510 | 48.76133333 | -98.83675 | LL+ | MS | 100 | 15 |  |
| 511 | 48.7905 | -98.90158333 | Null | MS | 100 | 0.04 |  |
| 512 | 48.79061667 | -99.00071667 | LL+ | MS | 100 | 0.08 |  |
| 513 | 48.79038333 | -99.09918333 | LL+ | MS | 100 | 0.07 |  |
| 514 | 48.79053333 | -99.1983 | LL+ | MS | 100 | 0.37 |  |
| 515 | 48.8052 | -99.27418333 | LL+ | MS | 100 | 0.22 |  |
| 516 | 48.85306667 | -99.3508 | RR + | MS | 100 | 0.08 |  |
| 517 | 48.8631 | -99.46085 | LL+ | MS | 100 | 0.3 |  |
| 518 | 48.86303333 | -99.56286667 | LL+ | MS | 100 | 0.82 |  |
| 519 | 48.84916667 | -99.65706667 | LL+ | MS | 100 | 0.01 |  |
| 520 | 48.838 | -99.74885 | Null | MS | 100 | 0.02 |  |
| 521 | 48.80853333 | -99.84091667 | RR + | MS | 100 | 0.36 |  |
| 522 | 48.80368333 | -99.9421 | Null | MS | 100 | 0.11 |  |
| 523 | 48.80606667 | -100.0382667 | LL+ | MS | 100 | 0.04 |  |
| 524 | 48.80635 | -100.13915 | Null | MS | 100 | 0.06 |  |
| 525 | 48.8064 | -100.2487333 | RR + | MS | 100 | 0.04 |  |
| 526 | 48.80855 | -100.3584 | Null | MS | 100 | 0.12 |  |
| 527 | 48.82086667 | -100.45075 | RR + | MS | 100 | 0.02 |  |
| 528 | 48.82103333 | -100.5667 | LL+ | MS | 100 | 0.09 |  |
| 529 | 48.82103333 | -100.67395 | RR + | MS | 100 | 0.04 |  |
| 530 | 48.82103333 | -100.7861667 | None | MS | 100 | 0 |  |
| 531 | 48.82095 | -100.88755 | None | MS | 100 | 0 |  |
| 532 | 48.82121667 | -101.001 | RR + | MS | 100 | 0.06 |  |
| 533 | 48.82113333 | -101.09675 | None | MS | 100 | 0 |  |
| 534 | 48.78845 | -101.1466833 | RR + | MS | 100 | 0.05 |  |
| 535 | 48.76295 | -101.21825 | LL+ | MS | 100 | 0.08 |  |
| 536 | 48.22895 | -101.2332667 | None | MS | 100 | 0 |  |
| 537 | 48.23925 | -101.1120833 | None | MS | 100 | 0 |  |
| 538 | 48.23963333 | -101.0035833 | LL+ | MS | 100 | 0.04 |  |
| 539 | 48.23958333 | -100.90435 | RR + | MS | 100 | 0.08 |  |
| 540 | 48.27346667 | -100.8146833 | RR + | MS | 100 | 0.07 |  |
| 541 | 48.2898 | -100.71875 | None | MS | 100 | 0 |  |
| 542 | 48.30711 | -100.61705 | Null | MS | 100 | 0.01 |  |
| 543 | 48.31165 | -100.5180667 | LL+ | MS | 100 | 0.02 |  |
| 544 | 48.31163333 | -100.4203333 | LL+ | MS | 100 | 0.01 |  |
| 545 | 48.23368333 | -101.3774333 | RR + | MS | 100 | 0.11 |  |
| 546 | 48.282 | -101.43545 | RR + | MS | 100 | 5.3 |  |
| 547 | 48.3086 | -101.5251 | LL+ | MS | 100 | 0.69 |  |
| 548 | 48.31303333 | -101.6398833 | None | MS | 100 | 0 |  |
| 549 | 48.32753333 | -101.7475667 | LL+ | MS | 100 | 0.03 |  |
| 550 | 48.32741667 | -101.8603333 | None | MS | 100 | 0 |  |
| 551 | 48.32735 | -101.9654167 | None | MS | 100 | 0 |  |
| 552 | 48.32578333 | -102.0781167 | None | MS | 100 | 0 |  |
| 553 | 48.32741667 | -102.1811167 | None | MS | 100 | 0 |  |
| 554 | 48.32708333 | -102.28945 | None | MS | 100 | 0 |  |
| 555 | 48.30745 | -102.39255 | RR + | MS | 100 | 0.19 |  |
| 556 | 48.31316667 | -102.5045333 | RR + | MS | 100 | 0.05 |  |
| 557 | 48.31308333 | -102.6064833 | RR + | MS | 100 | 0.05 |  |
| 558 | 48.31526667 | -102.7234167 | None | MS | 100 | 0 |  |
| 559 | 48.3422 | -102.8284 | RR + | MS | 100 | 0.06 |  |
| 560 | 48.34263333 | -102.9367 | None | MS | 100 | 0 |  |
| 561 | 48.34298333 | -103.044 | RR + | MS | 100 | 0.01 |  |
| 562 | 48.34263333 | -103.14735 | LL+ | MS | 100 | 0.01 |  |
| 563 | 48.3425 | -103.2491833 | None | MS | 100 | 0 |  |
| 564 | 48.34266667 | -103.3559667 | None | MS | 100 | 0 |  |
| 565 | 48.3429 | -103.4746333 | None | MS | 100 | 0 |  |
| 566 | 48.34256667 | -103.58335 | None | MS | 100 | 0 |  |
| 567 | 48.29421667 | -103.6260167 | None | MS | 100 | 0 |  |
| 568 | 48.22665 | -103.6260167 | None | MS | 100 | 0 |  |
| 569 | 48.16491667 | -103.6383 | None | MS | 100 | 0 |  |
| 570 | 48.14051667 | -103.7335333 | None | MS | 100 | 0 |  |
| 571 | 48.14041667 | -103.83925 | None | MS | 100 | 0 |  |
| 572 | 48.3158 | -101.5162167 | RR + | MS | 100 | 0.07 |  |
| 573 | 48.37235 | -101.5786167 | LL+ | MS | 100 | 0.05 |  |
| 574 | 48.41568333 | -101.6537833 | LL+ | MS | 100 | 0.14 |  |
| 575 | 48.4608 | -101.7426667 | RR + | MS | 100 | 0.19 |  |
| 576 | 48.48781667 | -101.8397833 | LL+ | MS | 100 | 0.11 |  |
| 577 | 48.53828333 | -101.9330333 | LL+ | MS | 100 | 0.24 |  |
| 578 | 48.54651667 | -102.0228833 | Null | MS | 100 | 0.11 |  |
| 579 | 48.54638333 | -102.1311833 | RR + | MS | 100 | 0.01 |  |
| 580 | 48.5464 | -102.2406 | LL+ | MS | 100 | 0.01 |  |
| 581 | 48.54728333 | -102.35585 | LL+ | MS | 100 | 0.02 |  |
| 582 | 48.56086667 | -102.4411333 | None | MS | 100 | 0 |  |
| 583 | 48.56106667 | -102.5601 | RR + | MS | 100 | 0.1 |  |
| 584 | 48.57511667 | -102.6553333 | None | MS | 100 | 0 |  |
| 585 | 48.57533333 | -102.7660167 | None | MS | 100 | 0 |  |
| 586 | 48.57546667 | -102.8648833 | None | MS | 100 | 0 |  |
| 587 | 48.59 | -102.9620667 | None | MS | 100 | 0 |  |
| 588 | 48.59003333 | -103.0706333 | None | MS | 100 | 0 |  |
| 589 | 48.57556667 | -103.1681667 | None | MS | 100 | 0 |  |
| 590 | 48.57551667 | -103.2765333 | None | MS | 100 | 0 |  |
| 591 | 48.58111667 | -103.3856 | None | MS | 100 | 0 |  |
| 592 | 48.57578333 | -103.4939833 | None | MS | 100 | 0 |  |
| 593 | 48.57091667 | -103.6025 | None | MS | 100 | 0 |  |
| 594 | 48.573 | -103.7114 | None | MS | 100 | 0 |  |
| 595 | 48.7633 | -101.3206333 | LL+ | MS | 100 | 0.09 |  |
| 596 | 48.76318333 | -101.4361833 | LL+ | MS | 100 | 0.05 |  |
| 597 | 48.7636 | -101.5405333 | None | MS | 100 | 0 |  |
| 598 | 48.7636 | -101.6592167 | LL+ | MS | 100 | 0.14 |  |
| 599 | 48.7636 | -101.7831167 | LL+ | MS | 100 | 0.01 |  |
| 600 | 48.76371667 | -101.884 | LL+ | MS | 100 | 0.01 |  |
| 601 | 48.76378333 | -101.9956333 | LL+ | MS | 100 | 0.04 |  |
| 602 | 48.77826667 | -102.08715 | RR + | MS | 100 | 0.02 |  |
| 603 | 48.80735 | -102.16075 | None | MS | 100 | 0 |  |
| 604 | 48.82651667 | -102.2396 | LL+ | MS | 100 | 0.31 |  |
| 605 | 48.8943 | -102.2566833 | Null | MS | 100 | 0.01 |  |
| 606 | 48.89443333 | -102.36335 | RR + | MS | 100 | 0.06 |  |
| 607 | 48.89445 | -102.4745167 | RR + | MS | 100 | 0.05 |  |
| 608 | 48.89446667 | -102.5838 | None | MS | 100 | 0 |  |
| 609 | 48.8944 | -102.6990333 | None | MS | 100 | 0 |  |
| 610 | 48.89463333 | -102.80825 | Null | MS | 100 | 0.17 |  |
| 611 | 48.89465 | -102.9181167 | None | MS | 100 | 0 |  |
| 612 | 48.89451667 | -103.0337667 | LL+ | MS | 100 | 0.16 |  |
| 613 | 48.89453333 | -103.1367167 | LL+ | MS | 100 | 0.01 |  |
| 614 | 48.8947 | -103.2463333 | Null | MS | 100 | 0.01 |  |
| 615 | 48.909 | -103.3557333 | None | MS | 100 | 0 |  |
| 616 | 48.90891667 | -103.4606833 | None | MS | 100 | 0 |  |
| 617 | 48.90901667 | -103.5750667 | None | MS | 100 | 0 |  |
| 618 | 48.90928333 | -103.68455 | None | MS | 100 | 0 |  |
| 619 | 48.90881667 | -103.8001 | RR + | MS | 100 | 0.03 |  |
| 620 | 48.06395 | -98.7841 | None | MS | 100 | 0 |  |
| 621 | 48.0498 | -98.68146667 | None | MS | 100 | 0 |  |
| 622 | 48.04973333 | -98.59335 | Null | MS | 100 | 0.02 |  |
| 623 | 48.03531667 | -98.45401667 | LL+ | MS | 100 | 0.09 |  |
| 624 | 48.03531667 | -98.35235 | None | MS | 100 | 0 |  |
| 625 | 48.02785 | -98.23371667 | None | MS | 100 | 0 |  |
| 626 | 48.0209 | -98.13941667 | LL+ | MS | 100 | 0.05 |  |
| 627 | 48.01773333 | -98.03211667 | LL+ | MS | 100 | 0.01 |  |
| 628 | 48.0052 | -97.93178333 | Null | MS | 100 | 0.04 |  |
| 629 | 48.00108333 | -97.82298333 | None | MS | 100 | 0 |  |
| 630 | 47.96228333 | -97.76208333 | Null | MS | 100 | 0.05 |  |
| 631 | 47.94685 | -97.6453 | None | MS | 100 | 0 |  |
| 632 | 47.93941667 | -97.53491667 | None | MS | 100 | 0 |  |
| 633 | 47.93271667 | -97.43623333 | RR + | MS | 100 | 0.03 |  |
| 634 | 47.93298333 | -97.32666667 | RR + | MS | 100 | 0.02 |  |
